# Preliminary Studies on Changes in Static Plantar Pressure and Stabilometry in Patients with Ankylosing Spondylitis Undergoing an Exercise Program

**DOI:** 10.3390/jcm13164673

**Published:** 2024-08-09

**Authors:** Ioana Gabriela Seres, Andrei Daniel Bolovan, Daniela Dragomir, Adina Octavia Duse, Daniel Popa, Georgeta Mioara Sinmarghitan, Elena Amaricai

**Affiliations:** 1 Doctoral School, “Victor Babes” University of Medicine and Pharmacy, 300041 Timisoara, Romania; ioana.handrea@umft.ro; 2 Municipal Clinical Emergency Hospital, 300254 Timisoara, Romania; dr.dragomir.daniela@gmail.com (D.D.); duse.adina@umft.ro (A.O.D.); popa.daniel@umft.ro (D.P.); sinmarghitan.georgeta@umft.ro (G.M.S.); 3 Department of Rehabilitation, Physical Medicine and Rheumatology, Faculty of Medicine,“Victor Babes” University of Medicine and Pharmacy, 300041 Timisoara, Romania; amaricai.elena@umft.ro; 4 Research Center for Assessment of Human Motion, Functionality and Disability, “Victor Babes” University of Medicine and Pharmacy, 300041 Timisoara, Romania

**Keywords:** ankylosing spondylitis, plantar pressure, stabilometry, physical exercise

## Abstract

**Background/Objectives**: Studies have reported that patients suffering from ankylosing spondylitis (AS) have decreased postural stability in comparison to healthy subjects. Our study aims to compare static plantar pressure and stabilometry parameters in AS patients who performed an 8-week exercise program (spine motion and flexibility exercises; stretching of hamstring, erector spine, and shoulder muscles; control abdominal and diaphragm breathing exercises and chest expansion exercises), in three different testing conditions (eyes open, eyes closed, and head retroflexed). **Methods**: Plantar pressure (the loading of the first and fifth metatarsal heads (MT1, MT5) and calcaneus) and stabilometry (CoP path length, 90% confidence ellipse area, and maximum CoP speed) were recorded in 28 AS patients (age 56.64 ± 10.3 years; body mass index 29.4 ± 4.9 kg/m^2^) at the beginning of rehabilitation and after 8 weeks. At first evaluation, there were significant differences (*p* < 0.05) for the foot loading sites (MT1, MT5, and calcaneus), both for the right and left feet, when comparing eyes open with the other two testing situations. **Results**: After rehabilitation, significant differences were recorded between eyes-open and head-retroflexed conditions for MT1 (*p* = 0.03 for right; *p* = 0.004 for left) and calcaneus (*p* = 0.014 for right; *p* = 0.011 for left). A significantly higher CoP path length was registered in both initial and final assessments when tested with eyes closed. The maximum CoP speed had increased values at both evaluations when tested with head retroflexed. **Conclusions**: The CoP path length decreased after the physical exercise program, with a better postural stability after rehabilitation.

## 1. Introduction

Ankylosing spondylitis (AS) is a chronic inflammatory disease of the sacroiliac joints and spine. Spinal stiffness typically ascends the spine over certain years, with movement restriction and spinal pain. Clinical features include the disappearance of lumbar lordosis, reduction in respiratory excursion due to the involvement of the costovertebral joints, and limitation of neck movement secondary to cervical spine inflammatory changes [1]. Other postural changes may occur, including cervical flexion, posterior rotation of the pelvis, hip extension, knee flexion, and plantar flexion of the ankle [2]. These changes can have a negative impact on balance. In advanced phases of the disease, patients have a typical kyphotic posture, which causes the displacement of the body’s center of gravity in the sagittal plane [3,4,5]. Other parts of the body develop compensatory changes to maintain the center of gravity within the limits of the supporting surface. When spinal ankyloses occur, only the mobile joints of the lower extremities can compensate for the displacement of the center of gravity. Durmus et al. [6] found that postural stability decreased during the early stages in patients with AS compared to unaffected individuals in addition to the more marked changes in the later stages. Russel [7] also noted a loss of balance in AS patients; poor balance was connected to severe joint deformities and falls. The study of Batur et al. [8] showed that in advanced stages, an increase in kyphosis causes impairments in anteroposterior stability and altered balance. 

Pedobarography is a method used to assess how the foot interacts with the supporting surface, can be used to measure both static and dynamic foot pressure, and is helpful for analyzing the biomechanics of a person’s walking and posture. When used in a static setting, pedobarography can measure the pressure exerted on the soles of the feet when standing. Plantar pressure assessments provide valuable data about postural control, as the feet play a crucial role in bearing weight and maintaining balance [9,10]. Postural control represents stability during standing and locomotion and the performance of voluntary tasks [11].

Stabilometry is an important tool for assessing patients with functional disorders and is commonly used in clinical practice to study human balance. Stabilometry quantifies the body sway of subjects in a standing position using a force platform [12]. This technique is based on the analysis of the time-variant center-of-pressure (CoP) coordinates [13]. The assessment of the variability in CoP is the most common technique used to quantify postural control in an upright stance. Nagymate and Kiss [14,15] studied correlation and variance analysis of CoP parameters in different standing conditions. The authors recommended the time-distance parameters as independent CoP parameters that are sufficiently sensitive to show the differences between different standing conditions. 

The usual treatment for AS involves a combination of anti-inflammatory drugs and exercise. Exercise seems to be more effective for this type of arthritis compared to others, with recommendations featuring prominently in relevant clinical guidelines for managing ankylosing spondylitis. However, specific information to guide exercise planning is lacking in clinical practice. Most of the published evidence focuses on mobility exercises, with relatively little attention being paid to other aspects of exercise program design such as strengthening and balance despite recognition that ankylosing spondylitis can affect muscle strength and balance [16,17,18].

The optimal mode of delivering treatment, along with the frequency and duration of the exercise program, needs to be thoroughly investigated. It is particularly important to determine whether specific components of exercise modalities can improve clinical outcomes. Some studies have shown that exercises have a small but significant positive effect on pain, spinal mobility, physical function, and overall patient assessment [19,20]. There is no clear evidence that exercise programs can cause adverse effects. However, it is important to note that severe adverse events are rare but can happen, such as falling. Exercise programs are generally associated with minor adverse effects: muscle or joint pain and soreness [21]. Based on the available evidence, it can be reassuring for people with AS to know that they can safely participate in common exercise programs, whether at home or in supervised facilities.

The objective of our study was to compare the static plantar pressure and stabilometry parameters in AS patients who performed an 8-week exercise program in different testing conditions (with eyes open, with eyes closed, and with head retroflexed). We hypothesized that there would be differences in the situations mentioned above, as well as before and after the physical exercise program.

## 2. Materials and Methods

### 2.1. Participants

**Figure 1:**
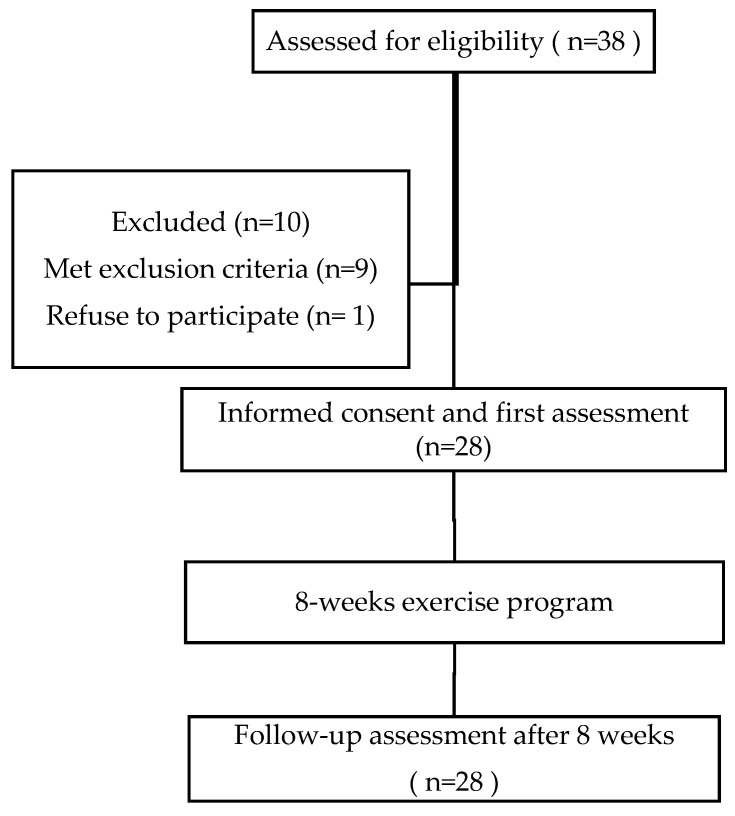
Flow chart of the study design.

In total, 28 patients with definite radiographic ankylosing spondylitis according to the *Assessment of Spondyloarthritis International Society 2009* criteria [22] who met the inclusion criteria were recruited for this study from the Rehabilitation and Rheumatology Department of the University County Hospital Timisoara, Romania, by personal invitation on one of our routine visits (Figure 1). Exclusion criteria consisted of medical conditions that impaired function more than the ankylosing spondylitis, such as the following: orthopedic pathologies of the lower limbs or history of orthopedic surgery of the lower limbs (that may impair limbs function, plantar pressure distribution, or stabilometry), foot involvement of AS, current complaints of foot pain, psychiatric disorders (dementia or other disorders that affect rationality), neurological diseases (stroke, Parkinson’s disease, etc.), vestibular or visual disturbances, and cardiopulmonary disorders that could affect participation in a physical exercise program. Patients treated with disease-modifying antirheumatic drugs had to be on a stable dosage for at least three months. Medication was not altered during the study period. 

Baseline patients’ characteristics were collected as follows: age, body mass index, duration of ankylosing spondylitis, specific disease functioning scores (Bath Ankylosing Spondylitis Disease Activity Index, Bath Ankylosing Spondylitis Functional Index), and medication (Table 1).

This study was conducted between December 2023 and April 2024. Participation in this study was voluntary and written informed consent was obtained from all the participants. This study was conducted in accordance with the Helsinki Declaration and was approved by the Ethics Committee of the “Victor Babes” University of Medicine and Pharmacy in Timisoara (reference no. 33/23.11.2023).

The sample size was calculated using G*Power 3.1.9.7 (Heinrich-Heine-Universität, Düsseldorf, Germany) with the Wilcoxon signed-rank test (matched pairs) [23]. The effect size was 0.8, the type I error was α = 0.05, and the power was 0.95. In total, 20 participants represent the minimum sample size [24,25].

### 2.2. Assessment

The static plantar pressure was recorded by a PoData device (Chinesport, Udine, Italy). The equipment registers the body weight distribution on the right foot and the left foot [26].

The subjects were assessed barefoot in standing position, without moving or talking, for 20 s. They stood on the platform with their knees extended and upper extremities along the trunk. Their feet were positioned at an angle of 30° to each other, with a distance of 5 cm between the heels. The plantar pressure testing with the above-mentioned feet position provides a more comfortable balance control condition than the feet parallel and in contact with each other. When comparing the testing configurations (with feet forming an angle of 30° and heels 5 cm apart and with feet parallel and 15 cm apart), it was suggested that both are a clinical option when testing impaired subjects [27].

The measurement was invalid if the subject undertook any motion from the standing posture with their arms, lower extremities, or head; raised their heel from the platform, talked; or lost the balance from the standing position. Plantar pressure was measured on three anatomical areas, namely the first (MT1) and fifth metatarsal heads (MT5) and the calcaneus. These data were recorded for both the right foot and the left foot [28]. 

For each of these sites, the percentage of body weight distribution was computed. An ideal load of an ideal subject was allocated as follows: 1/6 (16.67%) of the total weight on the fifth metatarsal head (MT5), 2/6 (33.33%) of the total weight on the first metatarsal head (MT1), and 3/6 (50%) of total weight on the heel [29].

The analyzed time-distance stabilometric parameters’ stabilometric data were CoP path length, 90% confidence ellipse area, and the maximum CoP speed, as recommended by Nagymate and Kiss [14]. 

The CoP path length is the length in millimeters of the subject’s center of gravity shift during the test. The confidence ellipse area is the area (calculated in mm^2^) of the ellipse that comprises all the center-of-gravity points measured and transferred on a system of Cartesian axes with a confidence level of 90%. The maximum CoP speed is the average center-of-gravity shifting maximum speed in millimeters per second.

The static plantar pressure and stabilometry measurements were completed for each participant under the following situations: with open eyes, with eyes closed, and with head retroflexed; measurements were made at baseline and after performing the 2-week physical exercise program.

### 2.3. Physical Exercise Program

The rehabilitation consisted of an 8-week physical exercise program. The first 2 weeks were performed in the Rehabilitation Department for 10 sessions (5 days per week for 2 weeks, 40 min per session) under the supervision of a trained physical therapist. Afterward, the patients continued with a home exercise program that was performed 5 days per week, 40 min per session. 

The exercise program consisted of motion and flexibility exercises of the cervical, thoracic, and lumbar spine; stretching of the hamstring muscles, erector spine muscles, and shoulder muscles; and abdominal and diaphragm breathing control exercises and chest expansion exercises [30,31]. The exercises of the rehabilitation program are described in Table 2. 

### 2.4. Statistical Analysis 

All statistical analyses were conducted using GraphPad Prism 5.0 for Windows. Descriptive statistics were computed for all variables (mean and standard deviation). Before statistical applications, the normal distribution of values in this study was verified by the D-’Agostino–Pearson normality test. The intragroup data (plantar pressure and stabilometric data for the three different conditions, as well as stabilometric data for baseline and after physical exercise program assessments) were compared with the paired *t*-test. A *p*-value of less than 0.05 was considered statistically significant [32].

This study analyzed the data of the same group of AS patients in different testing situations (static plantar pressure and stabilometric parameters before and after physical exercise program). We did not have two unrelated, independent groups of patients. Therefore, the independent sample *t*-test was not an option for our study [33].

## 3. Results

For the baseline assessment, the static pressure load distributions in the three testing conditions are presented in Table 3. When comparing the eyes-open and eyes-closed conditions, there were significant differences for all three sites of the right foot and left foot, with no differences in the total foot load of the right foot and left foot, respectively. For the right foot, we recorded increased MT1 load (*p* = 0.0005) and MT5 load (*p* < 0.0001), as well as decreased heel load (*p* = 0.0001), for eyes-closed testing. For the left foot, increased MT1 load (*p* < 0.0001) and MT5 load (*p* < 0.0001) and decreased heel load (*p* < 0.0001) were noted for eyes-closed condition. When comparing the eyes-open and head-retroflexed conditions, there were significant differences for all three sites of the right foot and left foot, with no differences in the total foot load of the right foot and left foot, respectively. For the right foot, increased MT1 load (*p* < 0.0001) and MT5 load (*p* = 0.0002) and decreased heel load (*p* < 0.0001) were registered when testing with the head retroflexed. For the left foot, increased MT1 load (*p* = 0.0025) and MT5 load (*p* = 0.043) and decreased heel load (*p* = 0.004) were reported for head-retroflexed condition. When comparing the eyes-closed and head-retroflexed conditions, there were no significant differences for the right foot and left foot and the three sites (MT1, MT5, and calcaneus), except for left MT5 (decreased load in head-retroflexed condition, *p* = 0.022).

For the second assessment (after an 8-week physical exercise program), the static pressure load distributions in the three testing conditions are presented in Table 4. When comparing the eyes-open and eyes-closed conditions, as well as eyes-closed and head-retroflexed conditions, there were no significant differences for the right foot and left foot and the three sites (MT1, MT5, and calcaneus). When comparing the eyes-open and head-retroflexed conditions, there were significant differences for the right MT1 and left MT1 (increased load in the head-retroflexed condition) and right heel and left heel (lower load in head-retroflexed condition). 

Table 5 includes the stabilometric data in the three testing conditions at the baseline assessment. When comparing the eyes-open and eyes-closed conditions, there were significant differences in CoP path length (*p* < 0.0001), 90% confidence ellipse area (*p* < 0.0001), and maximum CoP speed (*p* < 0.0001), with higher values when tested with eyes closed. When comparing the eyes-open and head-retroflexed conditions, there were significant differences in CoP path length (increased values for eyes-open condition, *p* = 0.0019) and maximum CoP speed (higher speed for head-retroflexed condition, *p* = 0.0039). When comparing the eyes-closed and head-retroflexed conditions, there were significant differences in CoP path length (*p* < 0.0001) and 90% confidence ellipse area (*p* = 0.038); the increased values were recorded when tested with eyes closed.

Table 6 presents the stabilometric data in the three testing conditions after an 8-week physical exercise program. When comparing the eyes-open and eyes-closed conditions, there were significant differences in CoP path length (*p* = 0.0016), 90% confidence ellipse area (*p* = 0.033), and maximum CoP speed (*p* = 0.0004), with higher values when tested with eyes closed. When comparing the eyes-open and head-retroflexed conditions, there were significant differences in 90% confidence ellipse area (*p* = 0.0075) and maximum CoP speed (*p* < 0.0001); higher values were registered when assessed with head retroflexed. There were no significant differences in the stabilometric data when comparing the eyes-closed and head-retroflexed conditions.

We also compared the stabilometric data within the same testing condition (eyes open, eyes closed, and head retroflexed) at the two evaluations (baseline and after an 8-week physical exercise program). When tested with eyes open, CoP path length, 90% confidence ellipse area, and maximum CoP speed had decreased values after rehabilitation; however, the differences were not statistically significant (*p* = 0.13 for CoP path length; *p* = 0.817 for 90% confidence ellipse area; *p* = 0.083 for maximum CoP speed). When tested with eyes closed, the stabilometric parameters had lower values after an 8-week exercise program; no significant differences were recorded (*p* = 0.092 for CoP path length; *p* = 0.93 for 90% confidence ellipse area; *p* = 0.57 for maximum CoP speed). In the head-retroflexed condition, the AS patients had statistically significant reduced CoP path lengths after rehabilitation (*p* = 0.018); the other two stabilometric parameters also decreased, but without a significant difference (*p* = 0.48 for 90% confidence ellipse area; *p* = 0.08 for maximum CoP speed). 

Regarding the stabilometry assessment, comparisons within the same testing condition were performed before and after the exercise program. The decreased values of stabilometric parameters show that AS patients achieved an improved postural stability after rehabilitation.

## 4. Discussion

Our study assessed the static plantar pressure and stabilometry in AS patients in three different conditions. In addition, we tested these parameters before the starting of a physical exercise program and after 8 weeks. 

The idea of analyzing these data with eyes closed came from the study of De Nunzio et al. [34] that aimed to assess the standing upright control and evaluate how the visual inflow affects the achievement of posture balance in AS patients. In total, 12 male patients (mean age 50.1 ± 13.2 years) were tested on a baropodometric platform with eyes open and with eyes closed. The authors found out that without the support of visual input, the AS patients had an increased amplitude and velocity of CoP oscillation.

In our research on 28 patients (mean age 56.64 ± 10.3 years), we recorded a significantly higher CoP path length at both initial and final assessments when tested with eyes closed. This fact supports the finding that poor postural stability is recorded without visual input. 

In addition to the eyes-closed condition, our patients also underwent a test with head retroflexed. Compared to eyes-open testing, significantly higher values of stabilometric parameters were recorded (CoP path length at initial evaluation; 90% confidence ellipse area at final evaluation; maximum CoP speed at both evaluations). These data suggest reduced postural stability when assessed with the head in extension. When comparing the data before and after the physical exercise program, the CoP path length was significantly reduced, thus suggesting a positive impact of the exercise program on obtaining better postural stability when assessed with head retroflexed. 

To the best of our knowledge, the evaluation of static plantar pressure and stabilometry in three distinct conditions (eyes open, eyes closed, and head retroflexed) was not performed before in AS patients. In addition, we retested these patients after an 8-week physical exercise program. 

The study of Gokcen et al. [35] on 50 AS patients showed that static foot posture is impaired in these patients. The Foot Posture Index-6 assessed static foot posture including six anatomical evaluations (talar head palpation, supra- and infralateral malleolar curvature, calcaneal frontal plane position, prominence in the region of talonavicular joint, congruence of the medial longitudinal arch, and abduction/adduction of forefoot on the rearfoot). Indeed, 72% of patients had a neutral foot posture, 16% had a pronated foot posture, and 12% of patients had a supinated foot posture.

Mesci et al. [36] investigated the possible changes in plantar load distribution in AS patients. A foot pressure platform assessed 30 AS patients and 30 healthy controls. The percentage total plantar load distribution over the proximal and distal halves of the foot was computed. AS patients had less percentage pressure applied on the distal half of the foot versus controls. The mean ratio of the percentage pressure distribution over the proximal half of the foot to the percentage pressure distribution over the distal half of the foot was significantly greater in AS patients.

In our study, we recorded a reduced heel load in all three testing conditions in both initial and final assessments, except for the left heel load when analyzed at baseline with eyes open. The loading of the right and left heel was even lower when assessed with eyes closed and head retroflexed at both assessment moments. 

The study of Demontis et al. [37] on AS patients divided into two groups (rehabilitation group versus educational group) showed improvement in the rehabilitation group (supervised training and home-based rehabilitation program plus educational behavioral therapy) rather than in the educational one for balance parameters, especially those measured with closed eyes. 

We recorded in our study better postural stability (expressed only by CoP path length) after an 8-week exercise program for the head-retroflexed assessment. In contrast to the study of Demontis [37] (patients’ follow-up of 7 months), our patients followed a relatively short-term rehabilitation (8 weeks). Despite the different durations of the physical exercise programs, we can confirm that rehabilitation programs for AS patients have a beneficial effect on balance impairment.

The systemic and inflammatory nature of AS, chronic pain, and changes in the bones and ligaments of the spinal column may affect the paravertebral muscles. Atrophy of paravertebral muscles may cause a decrease in strength and endurance of trunk extensor muscles [38]. The deterioration of the neutral position of the spine in AS may impair the stabilization synergy between the core muscles [39]. 

Acar et al. [39] showed that AS has negative effects on core stability and balance. They found that static postural stability (the overall stability index, anteroposterior stability index, and mediolateral stability index) was significantly reduced in AS patients when compared to healthy controls. The authors stated that it would be beneficial to add core stability and balance training to rehabilitation programs addressed for patients suffering from AS.

We also strengthen the idea of training the postural stability in AS patients. In addition to AS specific physical exercises, these patients should improve their balance in everyday activities to reduce the risk of falls. The postural balance should be maintained not only in eyes-open conditions but also in different situations such as with the head retroflexed (for example, when trying to take some objects from a higher level) or with eyes closed. 

The practical implications of the current research are represented by the early detection of loss of postural balance in AS patients not only when assessed with eyes open but also when tested with eyes closed. Adding exercises for increasing postural stability is targeted for assuring every day and leisure activities in order to maintain an optimal quality of life in this category of patients.

A consensus-based recommendation regarding exercise for AS states that individual exercise prescription should be informed by a thorough and reproducible assessment that includes musculoskeletal and psychosocial factors. Besides strength or cardiorespiratory function, balance should be assessed [16].

The lack of a dynamic plantar pressure assessment and the comparison with healthy controls can be considered limitations of the current study. We are aware that the second evaluation (after an 8-week physical exercise program) is a relatively short-term analysis. We intend to compare the static plantar pressure and stabilometric parameters before starting the physical exercise program and after a longer period (6 months and one year). Another future study aims to compare these postural stability data in AS patients who will follow two different types of physical exercise programs.

## 5. Conclusions

The assessment of static plantar pressure in AS patients in different testing conditions showed significant differences, especially when comparing eyes open and eyes closed and eyes open and head retroflexed, respectively. The differences were noted only after the 2-week physical exercise program when comparing eyes-open and head-retroflexed conditions. We recorded lower values of stabilometric parameters after the physical exercise program, pointing out a better stability. The rehabilitation programs in patients suffering from AS should also include exercises for improving postural balance.

## Figures and Tables

**Table 1:** Baseline patients’ characteristics.

Characteristics	Results
Gender	
Males (%)	20 (71.4%)
Females (%)	8 (28.6%)
Age (years) *	56.64 ± 10.3
Height (cm) *	166.4 ± 9.3
Weight (kg) *	81.3 ± 13.6
BMI (kg/m^2^) *	29.4 ± 4.9
BASFI *	4.1 ± 2
BASDAI *	1.9 ± 0.7
Medication (%)	
NSAIDs (%)	12 (42.8%)
SSZ (%)	6 (21.4%)
Biologics (%)	4 (14.2%)
SSZ + biologics (%)	4 (14.2%)
NSAIDs + MTX (%)	2 (7.1%)
NSAIDs + SSZ + biologics (%)	2 (7.1%)

*: Data are presented as mean ± standard deviation; BMI: body mass index; BASFI: Bath Ankylosing Spondylitis Functional Index; BASDAI: Bath Ankylosing Spondylitis Disease Activity Index; NSAID: non-steroidal anti-inflammatory drugs; SSZ: Sulfasalazine; MTX: Methotrexate.

**Table 2:** The physical exercise program.

Mobility Exercises.	Stretching Exercises	Breathing Exercises
Lying on the back with the knees bent: − Raise the knees towards the shoulders (10 repetitions); − Lower the knees to one side allowing the trunk to rotate (5 repetitions to right side and 5 repetitions to the left); − Raise both arms towards the ceiling (10 repetitions). Sitting on a gym ball with a neutral spine position: − Turn the head to look over the shoulder and then tuck the chin in to give a double chin (5 repetitions to right side and 5 repetitions to the left).	In standing upright position, against the wall: − Stretch to open the chest, holding the stretching for 10 s, 5 repetitions. In quadruped position: − Keeping the elbows straight, slowly arch the back as high as possible and then lengthen the neck, keeping the nose parallel to the floor, and hollow the back; maintain the stretching of the trunk extensors for 10 s and then the stretching of the abdominal muscles for 10 s; 5 repetitions.	In standing position: − With legs apart, grasp a stick with both hands and raise it above the head in inspiration; in exhalation, the hands come down in front Seated on a chair: − With a pulley in the hands, the hands extended in front: in inhalation, the hands go to the sides; in exhalation back to the initial position. − Each breathing exercise was repeated 5 times; after the first 5 sessions, the repetitions increased with 1 exercise per session, reaching a total of 10 repetitions for every breathing exercise.

**Table 3:** Static pressure load distribution in the testing conditions (baseline assessment).

Variables	Eyes Open	Eyes Closed	Head Retroflexed	p^a^	p^b^	p^c^
Right foot (%)	50.00 ± 3.00	50.00 ± 3.98	49.64 ± 5.59	0.98	0.67	0.52
Right MT1 (%)	18.43 ± 5.98	21.29 ± 5.85	21.21 ± 6.38	**0.0005**	**<0.0001**	0.92
Right MT5 (%)	33.00 ± 5.93	35.79 ± 5.32	35.29 ± 5.22	**<0.0001**	**0.0002**	0.42
Right heel (%)	48.36 ± 9.48	42.93 ± 9.03	43.64 ± 9.64	**0.0001**	**<0.0001**	0.59
Left foot (%)	50.00 ± 3.00	50.00 ± 3.98	50.36 ± 5.59	0.97	0.67	0.52
Left MT1 (%)	18.79 ± 4.55	22.07 ± 5.70	21.79 ± 7.27	**<0.0001**	**0.0025**	0.81
Left MT5 (%)	27.57 ± 4.26	30.43 ± 3.66	28.79 ± 3.98	**<0.0001**	**0.043**	**0.022**
Left heel (%)	53.64 ± 6.87	47.36 ± 7.09	49.50 ± 9.87	**<0.0001**	**0.004**	0.24

Data are presented as mean ± standard deviation; MT1: first metatarsal head; MT5: fifth metatarsal head; p^a^ relates to the difference between eyes-open and eyes-closed conditions; p^b^ relates to the difference between eyes-open and head-retroflexed conditions; p^c^ relates to the difference between eyes-closed and head-retroflexed conditions. The bolded *p*-values indicate significant differences.

**Table 4:** Static pressure load distribution in the testing conditions (assessment after physical exercise program).

Variables	Eyes Open	Eyes Closed	Head Retroflexed	p^a^	p^b^	p^c^
Right foot (%)	52.07 ± 14.47	53.00 ± 14.71	50.93 ± 7.06	0.074	0.48	0.22
Right MT1 (%)	21.50 ± 7.87	21.71 ± 6.50	23.79 ± 9.73	0.75	**0.03**	0.075
Right MT5 (%)	33.71 ± 8.49	33.57 ± 8.08	35.21 ± 6.99	0.88	0.062	0.129
Right heel (%)	44.64 ± 13.26	44.71 ± 10.98	40.93 ± 13.23	0.96	**0.014**	0.089
Left foot (%)	47.93 ± 14.47	47.00 ± 14.71	49.07 ± 7.06	0.074	0.48	0.23
Left MT1 (%)	20.64 ± 8.32	21.36 ± 8.63	23.00 ± 10.46	0.161	**0.004**	0.063
Left MT5 (%)	31.36 ± 12.05	31.64 ± 12.14	30.50 ± 7.42	0.482	0.402	0.26
Left heel (%)	49.00 ± 16.25	47.50 ± 15.98	46.36 ± 15.60	0.081	**0.011**	0.33

Data are presented as mean ± standard deviation; MT1: first metatarsal head; MT5: fifth metatarsal head; p^a^ relates to the difference between the eyes-open and eyes-closed conditions; p^b^ relates to the difference between the eyes-open and head-retroflexed conditions; p^c^ relates to the difference between the eyes-closed and head-retroflexed conditions. The bolded *p*-values indicate significant differences.

**Table 5:** Stabilometric data in the testing conditions (baseline assessment).

Stabilometric Data	Eyes Open	Eyes Closed	Head Retroflexed	p^a^	p^b^	p^c^
CoP path length (mm)	523.3 ± 22.6	783.9 ± 12.8	338.5 ± 85.31	**<0.0001**	**0.0019**	**<0.0001**
90% confidence ellipse area (mm^2^)	121.6 ± 9.66	263.1 ± 31.9	177.9 ± 29.18	**<0.0001**	0.053	**0.038**
Maximum CoP speed (mm/s)	62.29 ± 19.71	95.57 ± 7.04	92.64 ± 33.71	**<0.0001**	**0.0039**	0.094

Data are presented as mean ± standard deviation; CoP: center of pressure; p^a^ relates to the difference between eyes-open and eyes-closed conditions; p^b^ relates to the difference between eyes-open and head-retroflexed conditions; p^c^ relates to the difference between eyes-closed and head-retroflexed conditions. The bolded *p*-values indicate significant differences.

**Table 6:** Stabilometric data in the testing conditions (assessment after physical exercise program).

Stabilometric Data	Eyes Open	Eyes Closed	Head Retroflexed	p^a^	p^b^	p^c^
CoP path length (mm)	264.8 ± 58.42	385.4 ± 95.43	295 ± 39.35	**0.0016**	0.27	0.064
90% confidence ellipse area (mm^2^)	115.9 ± 8.39	258.1 ± 13.97	161.6 ± 17.8	**0.033**	**0.0075**	0.16
Maximum CoP speed (mm/s)	52.86 ± 18.03	88.43 ± 33.39	76.21 ± 25.84	**0.0004**	**<0.0001**	0.74

Data are presented as mean ± standard deviation; CoP: center of pressure; p^a^ relates to the difference between eyes-open and eyes-closed conditions; p^b^ relates to the difference between eyes-open and head-retroflexed conditions; p^c^ relates to the difference between eyes-closed and head-retroflexed conditions. The bolded *p*-values indicate significant differences.

## Data Availability

The data presented in this study are available on request from the corresponding author (A.D.B).

## References

[1] Vand Der Heijde D., Klippel J.H., Stone J.H., Crofford L.J., White P.H. (2008). Ankylosing Spondylitis. Primer on the Rheumatic Diseases.

[2] Koca T.T., Göğebakan H., Koçyiğit B.F., Nacitarhan V., Yildir C.Z. (2019). Foot Functions in Ankylosing Spondylitis. Clin. Rheumatol..

[3] Davis J.C., Koopman W.J., More-Land L.W. (2005). Ankylosing Spondylitis. Arthritis and Allied Conditions.

[4] Khan M.A., Hochberg M.C., Silman A.J., Smolen J.S., Winblatt M.E., Weis-Man M.H. (2003). Clinical Features of Ankylosing Spondylitis. Rheumatology.

[5] Park Y.G., Goh T.S., Kim D.S., Jung S.J., Lee J.S. (2023). Relationships between Clinical Status and Gait Parameters in Ankylosing Spondylitis. Clin. Orthop. Surg..

[6] Durmus B., Altay Z., Ersoy Y., Baysal O., Dogan E. (2010). Postural Stability in Patients with Ankylosing Spondylitis. Disabil. Rehabil..

[7] Russell A.S., Klippel J.H., Dieppe P.A. (1994). Ankylosing Spondylitis: History. Rheumatology.

[8] Batur E., Karataş G. (2017). Do Postural Changes Affect Balance in Patients with Ankylosing Spondylitis?. J. Rehabil. Med..

[9] Orlin M.N., McPoil T.G. (2000). Plantar Pressure Assessment. Phys. Ther..

[10] Mesci E. (2023). Pedobarographic Evaluations in Physical Medicine and Rehabilitation Practice. Turk. J. Phys. Med. Rehabil..

[11] Horak F.B., Binder M.D., Hirokawa N., Windhorst U. (2009). Postural Control. Encyclopedia of Neuroscience.

[12] De Blasiis P., Caravaggi P., Fullin A., Leardini A., Lucariello A., Perna A., Guerra G., De Luca A. (2023). Postural Stability and Plantar Pressure Parameters in Healthy Subjects: Variability, Correlation Analysis and Differences under Open and Closed Eye Conditions. Front. Bioeng. Biotechnol..

[13] Kapteyn T.S., Bles W., Njiokiktjien Ç.J., Kodde L., Massen C.H., Mol J.M.F. (1983). Standardization in Platform Stabilometry Being a Part of Posturography. Agressologie.

[14] Nagymate G., Kiss R.M. Replacing Redundant Stabilometry Parameters with Ratio and Maximum Deviation Parameters. Proceedings of the 12th IASTED International Conference on Biomedical Engineering, BioMed 2016.

[15] Nagymáté G., Kiss R.M. (2016). Parameter Reduction in the Frequency Analysis of Center of Pressure in Stabilometry. Period. Polytech. Mech. Eng..

[16] Millner J.R., Barron J.S., Beinke K.M., Butterworth R.H., Chasle B.E., Dutton L.J., Lewington M.A., Lim E.G.S., Morley T.B., O’Reilly J.E. (2016). Exercise for Ankylosing Spondylitis: An Evidence-Based Consensus Statement. Semin. Arthritis Rheum..

[17] Zheng J., Zhang X., Liu S., Ye F., Ji S., Wen X. (2024). Exercise Therapy Research in Ankylosing Spondylitis-Induced Back Pain: A Bibliometric Study (2004–2023). Med. Sci. Monit..

[18] Luo Y., Chen Y., Yan X., Zhang L., Shang Y., Seo J.C. (2024). Effectiveness of Exercise Intervention in Relieving Symptoms of Ankylosing Spondylitis: A Network Meta-Analysis. PLoS ONE.

[19] Dagfinrud H., Kjeken I., Mowinckel P., Hagen K., Kvien T. (2005). Impact of Functional Impairment in Ankylosing Spondylitis: Impairment, Activity Limitation, and Participation Restrictions. J. Rheumatol..

[20] Regnaux J.-P., Davergne T., Palazzo C., Roren A., Rannou F., Boutron I., Lefevre-Colau M.-M. (2019). Exercise Programmes for Ankylosing Spondylitis. Cochrane Database Syst. Rev..

[21] Kroon F.P., van der Burg L.R.A., Buchbinder R., Osborne R.H., Johnston R.V., Pitt V. (2014). Self-management Education Programmes for Osteoarthritis. Cochrane Database Syst. Rev..

[22] Rudwaleit M., Van Der Heijde D., Landewe R., Listing J., Akkoc N., Brandt J., Braun J., Chou C.T., Collantes-Estevez E., Dougados M. (2009). The Development of Assessment of SpondyloArthritis International Society Classification Criteria for Axial Spondyloarthritis (Part II): Validation and Final Selection. Ann. Rheum. Dis..

[23] Holmes D.T. (2020). Statistical Methods in Laboratory Medicine. Contemporary Practice in Clinical Chemistry.

[24] Faul F., Erdfelder E., Buchner A., Lang A.-G. (2009). Statistical Power Analyses Using G*Power 3.1: Tests for Correlation and Regression Analyses. Behav. Res. Methods.

[25] Kang H. (2021). Sample Size Determination and Power Analysis Using the G*Power Software. J. Educ. Eval. Health Prof..

[26] PoDATA 3.0. https://www.chinesport.com/catalog/posture-analysis/podoscopes/03021-podata-3-0.

[27] Scoppa F., Gallamini M., Belloni G., Messina G. (2017). Clinical Stabilometry Standardization: Feet Position in the Static Stabilometric Assessment of Postural Stability. Acta Medica Mediterr..

[28] Gobbi G., Galli D., Carubbi C., Pelosi A., Lillia M., Gatti R., Queirolo V., Costantino C., Vitale M., Saccavini M. (2013). Assessment of Body Plantar Pressure in Elite Athletes: An Observational Study. Sport Sci. Health.

[29] (2014). Global Postural System, Manual GPS 5—Version 1.0.42. http://www.michelevicario.net.

[30] Butendieck R.R., Maya J.J., Frontera W.R., Silver J.K., Rizzo T.D. (2019). Ankylosing Spondylitis. Essentials of Physical Medicine and Rehabilitation: Musculoskeletal Disorders, Pain, and Rehabilitation.

[31] Shatzer M., Choi H. (2018). Physical Medicine and Rehabilitation Pocketpedia.

[32] Free Updates to Prism Windows 5.04 and Prism Mac 5.0f for Current Prism 5 Us-Ers. https://www.graphpad.com/support/prism-5-updates/.

[33] Lewsey J. (2006). Medical Statistics: A Guide to Data Analysis and Critical Appraisal. Ann. R. Coll. Surg. Engl..

[34] De Nunzio A.M., Iervolino S., Zincarelli C., Di Gioia L., Rengo G., Multari V., Peluso R., Di Minno M.N.D., Pappone N. (2015). Ankylosing Spondylitis and Posture Control: The Role of Visual Input. BioMed Res. Int..

[35] Gokcen N., Sariyildiz A., Coskun Benlidayi I. (2021). Static Foot Posture and Its Relation to Clinical Variables in Ankylosing Spondylitis. Int. J. Rheum. Dis..

[36] Mesci E., Mesci N., Içagasıoglu A. (2017). Effects of Ankylosing Spondylitis on Plantar Pressure Distribution. Proceedings of the Poster Presentations.

[37] Demontis A., Trainito S., Del Felice A., Masiero S. (2016). Favorable Effect of Rehabilitation on Balance in Ankylosing Spondylitis: A Quasi-Randomized Controlled Clinical Trial. Rheumatol. Int..

[38] Resorlu H., Savas Y., Aylanc N., Gökmen F. (2016). Evaluation of Paravertebral Muscle Atrophy and Fatty Degeneration in Ankylosing Spondylitis. Mod. Rheumatol..

[39] Acar Y., Ilçin N., Gürpinar B., Can G. (2019). Core Stability and Balance in Patients with Ankylosing Spondylitis. Rheumatol. Int..

